# MOSAIC: A Spectral Framework for Integrative Phenotypic Characterization Using Population-Level Single-Cell Multi-Omics

**DOI:** 10.64898/2026.02.10.705077

**Published:** 2026-02-12

**Authors:** Chang Lu, Yuval Kluger, Rong Ma

**Affiliations:** 1 Computational Biology & Biomedical Informatics Program, Yale University, New Haven, CT, USA; 2 Department of Pathology, Yale University School of Medicine, New Haven, CT, USA; 3 Program in Applied Mathematics, Yale University, New Haven, CT, USA; 4 Department of Biostatistics, Harvard T.H. Chan School of Public Health, Boston, MA, USA; 5 Eric and Wendy Schmidt Center, Broad Institute, Cambridge, MA, USA; 6 Department of Data Science, Dana-Farber Cancer Institute, Boston, MA, USA

**Keywords:** Single-cell multi-omics, Population-scale analysis, Spectral data integration, Differential connectivity, Patient stratification

## Abstract

Population-scale single-cell multi-omics offers unprecedented opportunities to link molecular variation to human health and disease. However, existing methods for single-cell multi-omics analysis are either cell-centric, prioritizing batch-corrected cell embeddings that neglect feature relationships, or feature-centric, imposing global feature representations that overlook inter-sample heterogeneity. To address these limitations, we present MOSAIC, a spectral framework that learns a high-resolution feature × sample joint embedding from population-scale single-cell multi-omics data. For each individual, MOSAIC constructs a sample-specific coupling matrix capturing complete intra- and cross-modality feature interactions, then projects these into a shared latent space via spectral decomposition. The joint feature × sample embedding defines each feature’s connectivity profile per sample, enabling two key downstream applications. First, MOSAIC introduces Differential Connectivity (DC) analysis, which identifies features exhibiting regulatory network rewiring across conditions even when their expression or abundance remains unchanged. Applied to a CITE-seq vaccination cohort, MOSAIC revealed rewiring of proliferation programs in activated T cells, highlighting a functional shift in STAT5B despite stable expression. Second, MOSAIC enables identification of biologically meaningful sample subgroups by isolating coherent multi-modal feature modules. Applied to an HIV+ prefrontal cortex cohort, MOSAIC uncovered a novel stress-driven neuronal subtype within HIV+ samples characterized by elevated protein synthesis without chromatin accessibility changes. MOSAIC provides a general-purpose framework for systems-level phenotypic characterization, offering novel biological insights from population-scale multi-omic studies.

## 1 Introduction

The proliferation of single-cell technologies has created a new frontier in biological research, with datasets of unprecedented complexity. Modern single-cell studies are expanding along two critical axes. The first is multi-modality: we can now simultaneously profile the transcriptome, epigenome, and proteome from the same cell, capturing a multi-layered view of cell states [1–7]. The second is population scale: studies now span dozens or even hundreds of individuals from large clinical cohorts and atlases, aiming to disentangle disease- or condition-driven heterogeneity from technical noise [8–12]. This dual complexity, both multi-modal and multi-sample, is essential for linking cellular phenotypes to their underlying drivers, whether disease states, genetic variation, or environmental exposures. Yet realizing this potential requires computational frameworks that can integrate diverse data modalities while explicitly accounting for the biological variation across individuals.

Current integrative frameworks can be broadly grouped by their primary analytical focus. The majority are cell-centric: their principal goal is to learn a unified, low-dimensional representation of all cells [13–21]. To do this, they harmonize information from all modalities while simultaneously correcting for sample-specific batch effects. These methods are powerful for tasks such as cell type annotation and trajectory inference across samples. However, by treating cells as the basic unit of integration, they use features as passive inputs and fail to explicitly model feature-level heterogeneity or capture how feature relationships vary across biological conditions. Conversely, feature-centric frameworks have been developed to explicitly model the rich structure of cross-modal feature interactions [22–25]. Within this domain, feature co-embedding strategies are particularly prevalent, utilizing shared latent spaces to link diverse modalities [26–28, 18, 29]. While these approaches move beyond a single-modality view, they typically generate a fixed, global feature embedding shared by all cells and all samples. This ’one-size-fits-all’ representation, however, fundamentally masks the patient-to-patient heterogeneity that is essential for clinical insight. Thus, a critical gap remains for a framework that is both feature-centric and sample-aware: we still lack a method that moves beyond a single global map to explicitly model the phenotypic state of every feature for every individual.

Here, we present MOSAIC (Multi-Omic Sample-wise Analysis of Inter-feature Connectivity), a novel spectral framework designed to fill this critical gap by learning a high-resolution feature × sample joint embedding. MOSAIC’s process is two-fold. First, it constructs a distinct joint multi-modality feature embedding for each individual in the cohort, built by modeling the intricate web of feature-feature relationships that defines that sample’s unique network structure. Second, it projects all of these individual-specific embeddings into a common latent space. The result is a powerful new data representation where a feature’s “state” is defined by its position relative to all other features, which captures its complete functional context. Because all the samples are pro jected into a shared space, the network topology of each feature can be directly compared across samples, providing a unique foundation for novel, systems-level downstream analyses.

First, MOSAIC is designed to move beyond conventional differential feature detection. While powerful, existing methods [30–33] primarily rely on marginal tests, defining a “differential feature” as one whose mean level (expression, accessibility, or abundance) changes between conditions. This framework overlooks a subtler mode of variation: the rewiring of regulatory networks. MOSAIC is uniquely suited to detect such events. By defining a feature’s “state” through its functional context, MOSAIC enables Differential Connectivity (DC) analysis which tests for statistically significant shifts in a feature’s embedding position between conditions. This allows us to identify features whose functional roles and network relationships with other features are altered even when their abundance remains stable, revealing profound regulatory changes invisible to conventional differential analysis methods.

Second, MOSAIC can be used to identify hidden patient subgroups. In clinical studies, patients often receive a single label based on one measurement, like ’HIV+’ or ’diabetic’ [12, 34]. But this label can obscure important biological differences. Patients with the same label may respond differently to treatment or have distinct disease outcomes. The key question is whether biologically meaningful subgroups exist within these seemingly uniform populations. Existing approaches [35–38] typically compute global patient similarity across all features and then cluster patients accordingly. But this ’global’ approach often fails because critical signals from small, disease-relevant pathways may be diluted by noise from thousands of irrelevant features. MOSAIC overcomes this limitation through its feature × sample embedding. Instead of using all features simultaneously, MOSAIC first groups multi-modal features into biologically coherent modules based on their cross-patient interactions. It then tests whether any module delineates distinct patient subgroups. By isolating and evaluating these modules individually, MOSAIC reveals biologically meaningful patient subtypes that existing global methods often overlook.

Through extensive numerical experiments, we validate the MOSAIC framework, demonstrating that its embeddings are robust and biologically coherent for both features and samples. We then apply MOSAIC to two systems-level downstream analyses. First, for DC analysis, we use comprehensive simulations to show that MOSAIC uniquely detects pure network rewiring—signals completely missed by standard expression-based methods. We then apply MOSAIC to a vaccination cohort [13] and show it reveals a novel rewiring signature in T cells. Second, we show that, with its unsupervised subgroup detection, MOSAIC identifies a new stress-driven neuronal subtype in HIV+ individuals [12]. Together, these results establish MOSAIC as a general analytical framework for advancing from single-cell, single-modality profiles to a systems-level understanding of phenotypic variation in population-scale, multi-omic studies.

## 2 Results

### 2.1 Overview of MOSAIC Framework

**Figure 1.**
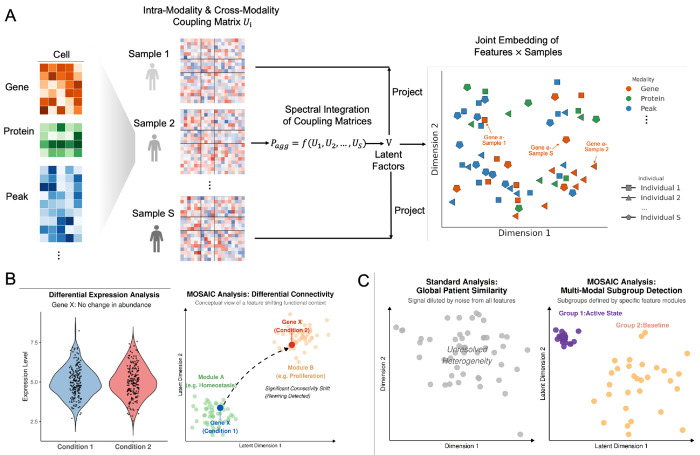
MOSAIC framework. **(A)** Schematic of the spectral integration approach: sample-specific coupling matrices (*U_i_*) are constructed from multi-omic data and integrated to learn latent factors (*V*), projecting features into a shared joint embedding space. **(B)** Differential Connectivity (DC) analysis detects features with network rewiring (right), identifying feature’s functional shifts distinct from standard differential abundance (left). **(C)** Unsupervised subgroup detection isolates coherent multi-modal feature modules to identify patient subtypes (right) often obscured in global similarity measures (left).

Conceptually, our framework (Figure 1A) operates in three stages. First, for each individual sample, we construct a sample-specific coupling matrix (*U_i_*) that captures the complete network of intra-modality and cross-modality feature-feature relationships unique to that individual. Second, we integrate these individual-specific matrices through a spectral decomposition to identify a common set of latent factors (*V*) shared across the population. Third, we pro ject each sample’s data onto these latent factors to generate the final joint embedding of features × samples (Methods).

The result is a novel data representation (Figure 1A, right) in which every feature (gene, chromatin peak, or protein) is represented by an embedding vector for each sample. This vector, defined relative to all other features in the space, captures the feature’s connectivity profile within that sample. Importantly, the same feature has different embedding vectors across samples. For example, in the embedding space, Gene A has one embedding vector in Sample 1 and different vectors in Sample 2 and Sample S, reflecting how its network relationships vary between individuals. This embedding structure provides a unified foundation for two powerful applications: Differential Connectivity analysis (Figure 1B) and unsupervised sample subgroup detection (Figure 1C).

#### MOSAIC Joint Embedding is Biologically Coherent and Technically Robust

To validate the MOSAIC framework, we used a public population-level, multi-omic cohort of human prefrontal cortex (PFC) tissue from people with HIV and controls [12]. The dataset consists of paired snRNA-seq and snATAC-seq data. For all analyses, we used gene expression from snRNA-seq and the accessibility of open chromatin regions (peaks) from snATAC-seq. Transcription factor (TF) information was derived from their RNA expression levels.

**Figure 2.**
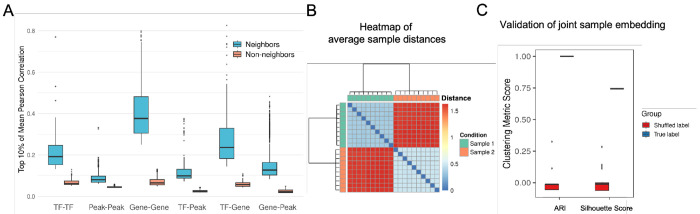
Biological coherence and technical robustness of MOSAIC feature × sample embeddings. **(A)** Aggregated top 10% mean Pearson correlations for feature pairs defined as neighbors (blue) versus non-neighbors (orange) in the MOSAIC joint embedding, across intra- and cross-modality comparisons. **(B)** Heatmap of average sample-to-sample distances across 100 subsampling iterations, showing distinct clustering by donor. **(C)** Clustering metrics (ARI and Silhouette Score) comparing subsample assignment to true labels (blue) versus shuffled labels (red).

We performed a series of technical validations to confirm that our feature × sample embedding is both biologically coherent and technically robust (Figure 2). For both validations, we used two representative samples from people with HIV in the PFC cohort. To validate biological coherence, we generated a joint feature embedding by averaging the feature × sample embeddings of these two individuals. We then tested whether features that are “neighbors” in this joint embedding space also share meaningful relationships in the original data from these same two samples (Figure 2A). We examined the feature embedding of each sample separately, then combined the results into a single plot. For each feature, we defined its ’Neighbors’ as the set of features closest to it in the joint embedding (Supplementary Methods). We returned to the original cell × feature data for the two samples, calculated a combined feature × feature Pearson correlation matrix, and computed the mean correlation for each feature against its ’Neighbors’ and a random set of ’Non-neighbors’. Across all intra- and cross-modality comparisons (e.g., TF-TF, Gene-Peak, TF-Gene), the correlation between neighbors was significantly higher than for non-neighbors. This confirms that features grouped together in the MOSAIC embedding are functionally related, demonstrating the biological coherence of our framework.

Second, we tested the technical robustness of the sample-specific embeddings using these same two samples (Figure 2B, C). A robust embedding should be a stable signature of an individual, not a random artifact of cell sampling. We performed a 100-iteration subsampling analysis: in each iteration, we randomly split each individual’s cells into 10 non-overlapping sub-samples, rebuilt the MOSAIC embeddings for each, and calculated the sample-to-sample distance between all 20 sub-samples (10 from each individual) using the Frobenius norm (Supplementary Methods). The results demonstrate perfect and highly stable separation. The averaged sample distance heatmap across 100 iterations consistently shows two distinct blocks, with all sub-samples from the same original donor clustering together (Figure 2B). To verify this separation reflects genuine biological signal rather than methodological artifacts, we compared clustering performance using true versus randomly shuffled sample labels across the 100 iterations (Figure 2C). True labels achieved near-perfect recovery of sample identity (ARI ≈ 1.0) and strong cluster cohesion (Silhouette ≈ 0.75) with minimal variance, while shuffled labels performed at chance level. This confirms that the MOSAIC feature × sample embedding is a highly robust and reproducible signature of an individual’s biology.

### 2.2 MOSAIC Differential Connectivity Analysis

While standard differential analysis methods focus on feature’s abundance shift, MOSAIC explicitly targets Differential Connectivity—the rewiring of a feature’s network partners independent of expression changes. The feature × sample embedding produced by MOSAIC is perfectly suited to detect such rewiring events. In our framework, a feature’s “state” is its functional context, defined by its position relative to all other features in the embedding space. We can therefore identify these DC features by directly testing for statistically significant shifts in a feature’s embedding position between conditions. To do this, we apply a Permutational Multivariate Analysis of Variance (PERMANOVA) test on the collection of a feature’s embedding vectors from all samples (Methods).

#### MOSAIC Uniquely Detects Differential Connectivity Events

To validate the specificity of our integrative approach and benchmark its performance as a true DC detector, we designed a comprehensive multi-modal simulation framework (Supplementary Methods). We generated data with three distinct signal types to systematically test MOSAIC against established abundance-based differential methods (DESeq2 [30], edgeR [31], MAST [32], and Seurat-Wilcoxon [39]): (1) Pure Connectivity Signal (Rewiring Only): Features had their underlying connectivity profile permuted between conditions, but their mean abundance was computationally rescaled to be identical, making this signal invisible to abundance-only methods and isolating connectivity changes alone. (2) Confounded Signal (Rewiring + Mean Shift): Features were rewired, and the resulting side-effect on mean abundance was not corrected. This represents a biologically common scenario where connectivity rewiring also produces a secondary mean expression shift. (3) Mean Shift Only: A classical differential expression signal where only feature abundance was changed, with no change in connectivity.

**Figure 3.**
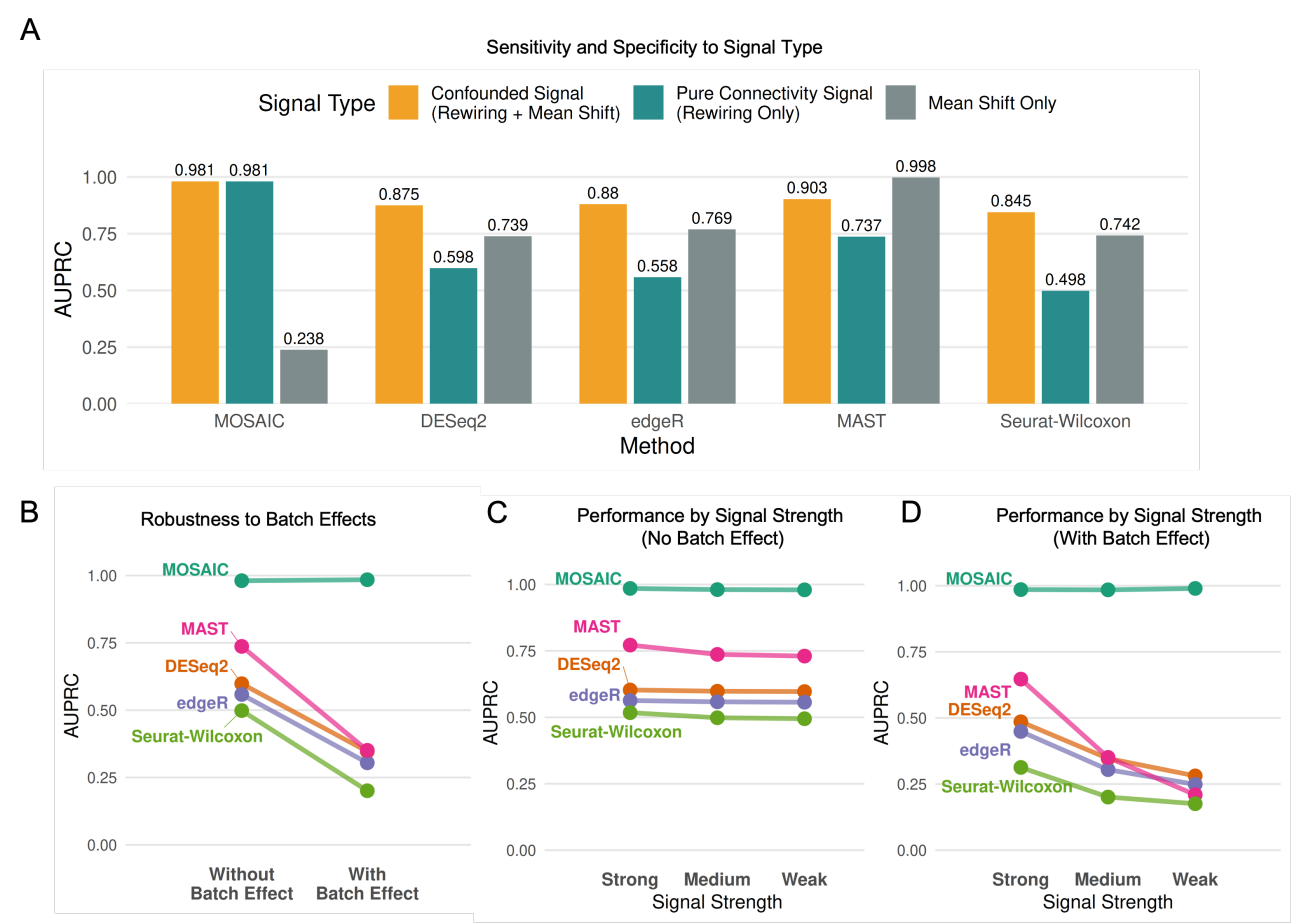
Benchmarking sensitivity and robustness. **(A)** AUPRC comparison across three simulation scenarios: Confounded Signal (Rewiring + Mean Shift), Pure Connectivity (Rewiring Only), and Mean Shift Only. MOSAIC detects pure connectivity changes missed by abundance-based methods. **(B)** Performance robustness (Slope graph) comparing methods with and without confounded batch effects. **(C–D)** Method performance with decreasing connectivity signal strength (Strong to Weak) in the absence **(C)** and presence **(D)** of batch effects.

The results in Figure 3A demonstrate MOSAIC’s unique and orthogonal capabilities. In the confounded signal scenario, where rewiring co-occurs with mean shifts, abundance-based methods achieved relatively high performance (e.g., Seurat-Wilcoxon AUPRC = 0.845), appearing to successfully detect differential signals. However, when we isolated pure connectivity changes by rescaling means to be identical, these same methods failed dramatically, with AUPRCs dropping to near random chance (e.g., Seurat-Wilcoxon AUPRC = 0.498). This stark contrast reveals that traditional methods detect mean-shift artifacts rather than genuine connectivity changes. Their apparent success in the confounded scenario was driven entirely by the secondary abundance signal. In contrast, MOSAIC maintained consistently high performance in both the confounded (AUPRC = 0.981) and pure connectivity (AUPRC = 0.981) scenarios, demonstrating its ability to specifically detect network rewiring independent of mean abundance changes. Critically, for the “Mean Shift Only” signal, abundance-based methods excelled (e.g., MAST AUPRC = 0.998), while MOSAIC correctly reported no significant connectivity changes (AUPRC = 0.238). This result provides essential evidence of MOSAIC’s orthogonality to traditional differential expression approaches—it is not a general-purpose abundance detector, but rather a specialized tool designed to identify network rewiring. Together, these results confirm that MOSAIC is not simply a proxy for abundance but is specifically and sensitively detecting connectivity changes by analyzing all modalities simultaneously.

#### MOSAIC Exhibits Robustness to Batch Effects and Signal Degradation

To demonstrate the practical utility of our approach, we evaluated its robustness against two common analytical challenges: technical batch effects and low signal-to-noise ratios.

First, we tested resilience to challenging, feature-specific batch effects in which the technical batch was confounded with the biological condition. As shown in Figure 3B, MOSAIC’s performance remained completely stable under strong batch effects (AUPRC ~ 0.98), while all competing methods showed significant degradation (e.g., DESeq2 AUPRC dropped from 0.598 to 0.347). This demonstrates the inherent robustness of the integrative approach to confounded batch effects. Next, we assessed sensitivity using simulations with progressively weaker connectivity signals (Strong, Medium, and Weak). As signal strength decreased, MOSAIC’s accuracy remained perfect regardless of signal strength, both without (Figure 3C) and with (Figure 3D) batch effects. In contrast, competing methods revealed a critical vulnerability: while their performance was relatively stable in an idealized, no-batch-effect setting (Figure 3C), the introduction of technical noise caused severe degradation (Figure 3D). As signal strength decreased from Strong to Weak in the presence of batch effects, traditional methods approached random chance, demonstrating that their sensitivity is not robust to the combined challenge of batch effect and signal degradation, where MOSAIC still remains highly stable.

Taken together, these simulations demonstrate that MOSAIC is a robust and highly specific framework for DC analysis. It is uniquely capable of identifying pure connectivity changes that are entirely missed by traditional abundance-based methods, while remaining exceptionally resilient to the technical confounders and signal degradation that compromise other approaches.

#### MOSAIC Uncovers a Rewired Proliferation and DNA Repair Program in Activated T Cells

To demonstrate MOSAIC’s utility on real-world data, we applied it to a multi-omic CITE-seq dataset (paired measurement of transcriptomes and cell-surface proteins) of human T-cells from 8 donors[13], comparing the naive state (Day 0) to the activated state (Day 7) post-vaccination. We focused our analysis on CD4+ Naive T-cells to isolate the specific signals of T-cell activation. MOSAIC identified 393 unique DC features (p-adj < 0.05), the vast majority of which (392/393) were not found by traditional differential expression analysis by MAST. This indicates that our method uncovers a completely distinct layer of biological change. To validate that these unique hits were biologically meaningful and not random noise, we performed three complementary global analyses.

**Figure 4.**
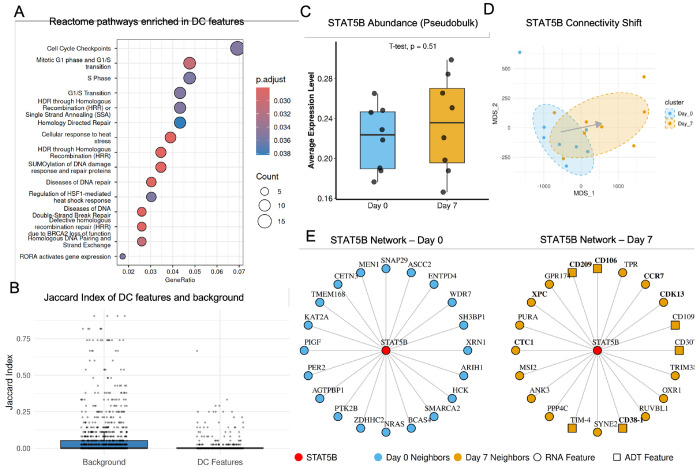
Rewiring of proliferation networks in activated T cells. **(A)** Reactome pathway enrichment of 393 DC features. **(B)** Jaccard Index of neighbor overlap (Day 0 vs. Day 7) for DC features versus background, showing higher turnover in DC features (*p* = 4.3 × 10^−15^). **(C)** Box plot of pseudobulk expression (average per sample) showing no significant difference between Day 0 and Day 7 (*p* = 0.51, T-test). **(D)** MOSAIC embedding shift of STAT5B from Day 0 (blue) to Day 7 (orange), showing systematic shift in the mean connectivity profile. **(E)** STAT5B’s top 20 connectivity partners at Day 0 (left) and Day 7 (right), showing network topology turnover.

First, Reactome pathway analysis [40] revealed that the 393 DC features are significantly enriched in pathways central to T-cell clonal expansion (Figure 4A). The top enriched terms delineate a coordinated program spanning “Cell Cycle Checkpoints” and the “G1/S Transition,” alongside critical DNA repair mechanisms such as “HDR through Homologous Recombination.” This confirms that the features rewired during activation represent the core machinery linking rapid cell proliferation with the genome integrity mechanisms necessary to support it. Second, protein-protein interaction network analysis using STRING revealed that the DC proteins showed significant enrichment for known physical and functional interactions (493 interactions observed vs. 392 expected, *p* = 5.56*e* – 07; Figure S1). This network enrichment indicates that the rewired features are not randomly distributed but form an interconnected regulatory network, providing additional evidence that MOSAIC identifies functionally coordinated sets of features undergoing concerted reorganization during T-cell activation. Third, we quantitatively validated that DC features exhibit significantly higher network turnover than background features across condition. We measured the stability of each feature’s functional context by calculating the Jaccard Index of its neighbor overlap between the naive (Day 0) and activated (Day 7) states (Figure 4B). The DC features showed significantly lower Jaccard scores (*p* = 4.3 × 10^−15^), providing statistical evidence that their regulatory neighborhoods undergo substantially greater reorganization compared to the global background.”

To illustrate these findings at a mechanistic level, we investigated the STAT5B, a canonical T-cell transcription factor identified by our method (DC rank 93, p-adj = 0.015). Traditional methods missed this key regulator, as its gene expression level remained stable between Day 0 and Day 7 (Figure 4C). MOSAIC, however, detected a highly significant shift in its connectivity profile, visualized by the clear separation of its sample embeddings (Figure 4D), with an arrow indicating the systematic shift from the Day 0 centroid to the Day 7 centroid. To interpret the biological meaning of this rewiring, we examined the top 20 connectivity partners of STAT5B (Figure 4E). The network exhibited a complete turnover in partners between the two timepoints, with no overlap. The Day 0 network consisted of general regulators, such as the chromatin remodeler SMARCA2 and the signaling protein NRAS. In contrast, the Day 7 network was rewired to features directly controlling the activated T-cell phenotype. We found new connections to the canonical trafficking receptor CCR7 [41] and the classic activation marker CD38-1 [42], as well as numerous other surface proteins like CD106 and CD209. Notably, this rewiring directly links STAT5B to the proliferation machinery discovered in our pathway analysis (Figure 4A). New Day 7 partners include the cell-cycle regulator CDK13 [43] and the DNA repair proteins XPC and CTC1 [44, 45]. This aligns with recent evidence that STAT5B phosphorylation is strictly required to induce the expression of cell cycle regulators necessary for maximal T cell expansion [46]. Moreover, the concurrent recruitment of DNA repair factors reflects the critical biological requirement to maintain genomic integrity during the replication stress of rapid cell division [47]. Together, these results demonstrate that STAT5B, while not changing in abundance, has completely rewired its functional context from a basal regulatory state to one that is poised to orchestrate T-cell activation, trafficking, and proliferation—a multi-omic functional transition that is invisible to traditional abundance-based methods.

### 2.3 MOSAIC Enables Unsupervised Multi-Modal Sample Subgroup Detection

We next applied MOSAIC to dissect latent patient heterogeneity. Unlike global clustering methods that can be obscured by noise from irrelevant features, MOSAIC utilizes a modular strategy to identify robust patient subgroups. Specifically, we leverage the joint embedding to calculate a stratification profile for each feature, capturing how it distinguishes samples from one another. By clustering these profiles, MOSAIC identifies feature modules, which are groups of features that co-vary similarly across the cohort (Methods). This allows us to move beyond a single global map and evaluate whether specific molecular modules drive distinct, biologically meaningful patient subgroups.

#### Validation of MOSAIC Subgroup Detection on HIV and Control Cohorts

**Figure 5.**
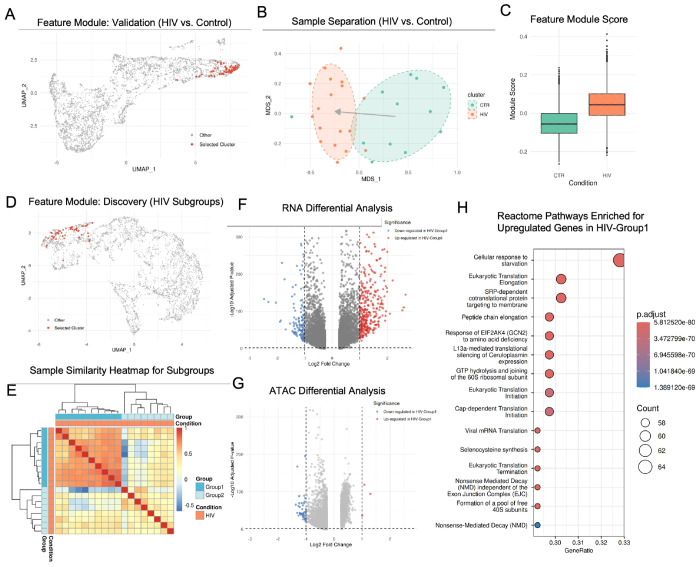
**(A–C)** Validation using L2/3 inhibitory neurons HIV+ and Control samples: **(A)** Feature module (red) identified to validate the separation of HIV+ and control samples. **(B)** MOSAIC sample embeddings based on the module in A, showing clear separation of the HIV+ and control samples. **(C)** Feature module scores in HIV+ vs. Control. **(D–H)** Discovery of HIV subgroups: **(D)** Feature module (red) identified within the HIV+ cohort. **(E)** Sample similarity heatmap based on the discovery feature module from panel D, partitioning HIV+ samples into a coherent HIV-Group1 and a heterogeneous HIV-Group2. **(F)** RNA differential expression and **(G)** ATAC differential accessibility between the two HIV subgroups. **(H)** Reactome enrichment for genes upregulated in HIV-Group 1.

To first validate this approach, we applied it to L2/3 inhibitory neurons from the multi-omic prefrontal cortex cohort of 30 donors (18 HIV+, 12 controls) with paired snRNA and snATAC measurements [12], treating all disease labels as unknown. We sought to test whether MOSAIC could successfully identify the subgroup structure distinguishing HIV+ samples from controls without prior knowledge. Applying the feature-clustering workflow described above, the analysis successfully identified a prominent feature module (Figure 5A). The sample embedding based on this module shows clear and significant separation between the two cohorts (Figure 5B). To validate that this module captures true biological signal rather than technical artifacts, we calculated module scores for each cell from the original single-cell data (Supplementary Methods). The distribution of module scores differed significantly between cells from HIV+ and control samples (Figure 5C), showing a clear increase in the HIV+ cohort and confirming that the identified feature module reflects genuine HIV-associated effects in this neuronal population. Together, these results validate that MOSAIC’s feature-centric approach can robustly identify primary biological structure in complex datasets without supervision.

#### MOSAIC Discovers a New Stress-Driven Neuronal Subtype in HIV+ Patients

The true power of this approach lies in discovering previously unknown patient subtypes hidden within diagnostic labels. To demonstrate this capability, we applied MOSAIC’s unsupervised clustering exclusively to the 18 HIV+ samples. This analysis revealed a robust feature module (Figure 5D) that partitioned the HIV+ cohort into two distinct, previously unrecognized subgroups. This cryptic structure is clearly visible in the sample similarity heatmap (Figure 5E). Based solely on this feature module, the samples were clustered into two groups: HIV-Group1 (n=10) forms a tight, highly coherent cluster, while HIV-Group2 (n=8) appears more heterogeneous. This structure suggests that the feature module has identified a common biological state shared by patients in Group 1 that is absent or variable in Group 2.

To validate this hypothesis and characterize this putative state, we performed differential analysis between the two groups. The results confirmed a strong, RNA-dominated biological shift. The RNA volcano plot (Figure 5F) shows a dramatic asymmetry, with numerous genes significantly upregulated in HIV-Group1 compared to HIV-Group2. In contrast, this massive gene-level signal was not mirrored at the chromatin level, where we observed very few significantly differential ATAC features (Figure 5G). This confirms that Group 1 is defined by the gain of a specific transcriptional program that is absent in Group 2. To define the functional identity of this state, we performed Reactome pathway enrichment analysis on the upregulated genes in HIV-Group1. The results (Figure 5H) revealed a dominant signature of translational machinery operating under metabolic stress. The profile was driven by the top term “Cellular response to starvation,” alongside a broad upregulation of core protein synthesis components (e.g., translation elongation and SRP-dependent targeting). Notably, the specific enrichment of GCN2-mediated signaling indicates activation of the Integrated Stress Response (ISR). This molecular profile implies that HIV-Group1 neurons are undergoing chronic metabolic exhaustion and proteostatic stress, a known feature of HIV-associated neurocognitive disorders (HAND) that compromises neuronal function [48, 49]. The paradoxical upregulation of translation machinery in this context may reflect a compensatory effort to maintain synaptic plasticity despite the neurotoxic environment [50].

Together, these findings demonstrate that MOSAIC identified a biologically coherent subgroup of HIV+ samples within L2/3 inhibitory neurons defined by a specific stress-response endotype. By focusing on modular connectivity rather than global similarity, MOSAIC revealed a translation-driven, ISR-like state that would likely remain invisible to standard clustering approaches.

## 3 Discussion

### Differential Connectivity as an Orthogonal Axis of Variation

A central contribution of this work is formalizing Differential Connectivity (DC) as a distinct biological metric. While standard differential expression identifies changes in feature abundance, it fundamentally overlooks network rewiring—where a feature’s regulatory context shifts despite stable expression. Our results confirm that DC is not merely a proxy for expression changes but an orthogonal signal; MOSAIC successfully identified key regulatory drivers (e.g., STAT5B) that were invisible to abundance-based metrics. By capturing these hidden functional transitions, MOSAIC allows researchers to dissociate a feature’s “state” (abundance) from its “function” (connectivity), offering a more complete view of cellular regulation.

### Modular vs. Global Patient Stratification

Beyond feature discovery, MOSAIC introduces a modular paradigm for patient stratification. Conventional clustering, which computes global patient similarity across all features, is prone to signal dilution when disease heterogeneity is driven by small, specific pathways. By instead deriving patient similarity from coherent feature modules, MOSAIC amplifies relevant biological signals while suppressing noise from invariant features. This targeted approach is critical for decomposing complex diagnostic labels into mechanistically distinct endotypes, as demonstrated by our identification of the stress-driven “cryptic” subgroup within the HIV+ cohort.

### Robustness via Spectral Network Abstraction

MOSAIC mitigates technical noise by abstracting data into second-order feature relationships. By converting raw expression values into correlation-based coupling matrices, our framework prioritizes relative data structure over absolute magnitudes. This effectively acts as a filter for technical variation: while batch effects often manifest as global shifts in mean expression magnitudes, local feature-feature correlations remain comparatively stable. Consequently, MOSAIC achieves “zero-configuration” robustness, maintaining high specificity in the presence of confounded batch effects without requiring the explicit correction parameters often needed by abundance-based models.

### Computational Complexity and Limitations

The rigorous modeling of feature relationships imposes specific computational demands. The construction of sample-specific coupling matrices scales as *O*(*S · n · F*^2^), where *S* is the number of samples, *n* is the average number of cells, and *F* is the number of features. The subsequent spectral decomposition scales as *O*(*S · F*^2^ · *k*) by utilizing a truncated eigendecomposition for the top *k* components. Thus, the framework is computationally linear with respect to sample size (making it scalable to large cohorts) but quadratic with respect to feature count. For extremely high-dimensional spaces (eg. *F* > 50, 000), this necessitates feature selection to prioritize informative variables. Furthermore, the current framework assumes paired multi-omic data. Extending MOSAIC to unpaired modalities would require upstream manifold alignment to infer cell-cell correspondences, utilizing computational frameworks such as UnpairReg [51].

### Conclusion

MOSAIC establishes a spectral framework for analyzing the relational structure of single-cell populations. By shifting the analytical lens from abundance to connectivity, it uncovers regulatory wiring that underpins cellular states but remains invisible to standard metrics. This approach provides a technically robust, mathematically grounded toolkit for dissecting the complex phenotypic heterogeneity that defines human health and disease.

## 4 Methods

### MOSAIC Framework

The MOSAIC framework is a multi-stage spectral method designed to learn a feature × sample joint embedding from population-level, multi-omic data. The overall workflow, summarized in Figure 1A, is described in detail below.

#### Input Structure and Data Preprocessing

Consider a cohort consisting of *S* samples and *K* data modalities (e.g., RNA, ATAC, Protein). For each sample *i* ∈ {1, …, *S*}, let the data from modality *k* be represented by a cell-by-feature matrix $X_{i}^{\left(k\right)}\in R^{n_{i}\times F_{k}}$, where *n_i_* denotes the number of cells in sample *i* and *F_k_* denotes the number of features in modality *k*. All input data are assumed to be normalized and scaled.

#### Construction of Sample-Specific Coupling Matrices

To establish a unified feature space for sample i, we first concatenate the modality-specific matrices horizontally: $X_{i}=\left[X_{i}^{\left(1\right)}|\ldots |X_{i}^{\left(K\right)}\right]\in R^{n_{i}\times F}$, where $F=\sum_{k=1}^{K}F_{k}$ is the total feature count. We then compute a sample-specific coupling matrix $U_{i}\in R^{F\times F}$ that captures intra-sample feature relationships. Specifically, the entry $u_{jl}^{\left(i\right)}$ corresponds to the cosine similarity between the j-th and l-th feature vectors (columns) of $X_{i}:u_{jl}^{\left(i\right)}=\frac{x_{i,j}\cdot x_{i,l}}{{\left\|x_{i,j}\right\|}_{2}{\left\|x_{i,l}\right\|}_{2}}$. Thus, $U_{i}$ represents the complete, normalized inter-feature connectivity network topology for sample *i*.

#### Spectral Integration and Latent Factor Generation

To identify global latent factors shared across the population, for each sample i, we perform eigendecomposition $U_{i}=V_{i}\Lambda_{i}V_{i}^{T}$, where $V_{i}\in R^{F\times F}$ and $\Lambda_{i}$ is diagonal. By retaining the top $r_{i}$ eigenvectors, we obtain the low-rank approximation $U_{i}^{\left(r_{i}\right)}=V_{i}^{\left(r_{i}\right)}\Lambda_{i}^{\left(r_{i}\right)}{\left(V_{i}^{\left(r_{i}\right)}\right)}^{⊤}$, effectively denoising the sample-specific networks. We then aggregate these approximations into a global projection matrix $P_{\text{agg}}=\sum_{i=1}^{S}U_{i}^{\left(r_{i}\right)}$. A final eigendecomposition of $P_{\text{agg}}=V_{agg}\Lambda_{agg}V_{agg}^{T}$ and select top d eigenvalues yields the final consensus embedding $V=V_{agg}^{\left(d\right)}\Lambda_{agg}^{\left(d\right)}\in R^{F\times d}$. The dimensionality $r_{i}$ and d is determined adaptively using the Kneedle algorithm [52].

#### Generation of the Final Feature × Sample Embedding

For each sample i, we project the sample-specific connectivity network onto the global latent basis to obtain the final embedding $E_{i}=U_{i}V\in R^{F\times d}$. The collection of these embeddings forms a third-order tensor $ℰ\in R^{F\times d\times S}$, where the slice $e_{f}^{\left(i\right)}\in R^{d}$ represents the functional state of feature f in sample i aligned to the common population space.

### Differential Connectivity Feature Identification

The input consists of the final feature × sample embedding tensor $ℰ\in R^{F\times d\times S}$ and a vector of condition labels $c\in \left\{c_{1},\ldots ,c_{S}\right\}$ corresponding to the S samples.

#### Construction of Feature Trajectory Matrices.

For each feature *f*, we extract its corresponding slice from the tensor ℰ to form a feature-specific matrix $E_{f}\in R^{S\times d}$. The i-th row of this matrix, $e_{f}^{\left(i\right)}$, represents the embedding coordinates of feature *f* in sample *i*. This matrix effectively captures the phenotypic tra jectory of a single feature across the entire patient cohort.

#### Quantification of Geometric Shift.

To quantify rewiring, we compute a sample-to-sample Euclidean distance matrix $D_{f}\in R^{S\times S}$ based on the rows of $E_{f}$. We then apply a Permutational Multivariate Analysis of Variance (PERMANOVA) to test if the feature’s position shifts significantly between conditions. The magnitude of this shift is quantified by the pseudo-F statistic: $F=\frac{SS_{B}/(g-1)}{SS_{W}/(S-g)}$, where S is the total number of samples, g is the number of condition groups, $SS_{W}$ is the sum of squared distances within groups, and $SS_{B}$ is the sum of squared distances between groups. This statistic effectively measures the ratio of signal (condition-driven separation) to noise (within-group variability) for each feature.

#### Statistical Significance and FDR Control.

To rigorously distinguish true biological rewiring from random embedding artifacts, we compute empirical *p*-values using a global null model. Unlike standard implementations that permute the final distance matrix, we shuffle the sample labels of the original cells prior to the coupling matrix construction. We then re-compute the entire MOSAIC embedding on this permuted dataset. Since all condition-specific biological signals are destroyed in this null data, any resulting *F*-statistics represent the background noise distribution of the framework. We pool the *F*-statistics from all features in this null embedding to form a global null distribution. The empirical *p*-value for feature *f* is derived by calculating the quantile of its observed *F*-statistic within this null distribution. Features with an adjusted *p*-value < 0.05 are classified as Differential Connectivity features.

### Unsupervised Sample Subgroup Detection

The input is the final feature × sample embedding tensor $ℰ\in R^{F\times d\times S}$. This analysis proceeds in three steps: defining feature-specific stratification patterns, identifying coherent modules, and clustering patients based on those modules.

#### Construction of Feature Stratification Profiles.

We first quantify how each individual feature separates the patient cohort. For each feature f, we extract the sample embedding matrix $E_{f}\in R^{S\times d}$, where the i-th row corresponds to the embedding of feature f in sample i. We compute a feature-specific sample-to-sample distance matrix $D_{f}\in R^{S\times S}$ using cosine distance: $D_{ij}^{\left(f\right)}=1-\frac{e_{f}^{\left(i\right)}\cdot e_{f}^{\left(j\right)}}{{\left\|e_{f}^{\left(i\right)}\right\|}_{2}{\left\|e_{f}^{\left(j\right)}\right\|}_{2}}$. We vectorize the lower triangular portion of $D_{f}$ to create a vector $v_{f}\in R^{S(S-1)/2}$. This vector, termed the Stratification Profile, encodes the specific patient similarity structure dictated by feature f.

#### Identification of Coherent Feature Modules.

To find groups of features that drive similar patient stratifications, we construct a feature × feature similarity matrix $S_{\text{feat}}\in R^{F\times F}$. Each entry $S_{fg}$ is the Spearman rank correlation between the stratification profiles $v_{f}$ and $v_{g}$. We apply hierarchical clustering to $S_{\text{feat}}$ to identify feature modules—subsets of features ℳ⊂{1,…,F} that exhibit highly correlated stratification patterns across the cohort.

#### Module-Driven Patient Clustering.

For a selected feature module ℳ, we compute a final patient similarity matrix $S_{ℳ}\in R^{S\times S}$ to partition the cohort. The similarity between patients i and j is defined as the cosine similarity of their aggregated module embeddings: $S_{ij}^{\left(ℳ\right)}=cos\left(vec\left(E_{i}[ℳ,:]\right),vec\left(E_{j}[ℳ,:]\right)\right)$, where $E_{i}[ℳ,:]$ represents the submatrix of embeddings for features in ℳ for sample i, and vec(⋅) denotes the flattening of this matrix into a single vector. Hierarchical clustering on $S_{ℳ}$ reveals cryptic patient subgroups driven specifically by the biological process captured within module ℳ.

## Supplementary Material

1

## Data Availability

The human CITE-seq vaccination cohort data [13] is available from the Gene Expression Omnibus (GEO) under accession number GSE164378 or via the link: https://atlas.fredhutch.org/nygc/multimodal-pbmc/. The single-nucleus multi-omic prefrontal cortex (PFC) dataset [12] is available from the corresponding authors upon reasonable request.

## Code Availability

The source code for MOSAIC and usage examples are available on GitHub at https://github.com/KlugerLab/MOSAIC.
