# Supplementary material for: MOSAIC: A Spectral Framework for Integrative Phenotypic Characterization Using Population-Level Single-Cell Multi-Omics
Source: bioRxiv. 2026 Feb 27:2026.02.10.705077. Originally published 2026 Feb 12. Preprint. [Version 2] doi: 10.64898/2026.02.10.705077 (PMC12918988; doi:10.64898/2026.02.10.705077)
Supplement: 1 [file NIHPP2026.02.10.705077V2-supplement-1.pdf]

bioRxiv preprint doi: https://doi.org/10.64898/2026.02.10.705077; this version posted February 27, 2026. The copyright holder for this preprint (which was not certified by peer review) is the author/funder, who has granted bioRxiv a license to display the preprint in perpetuity. It is made available under aCC-BY 4.0 International license.

A Supplementary Figure

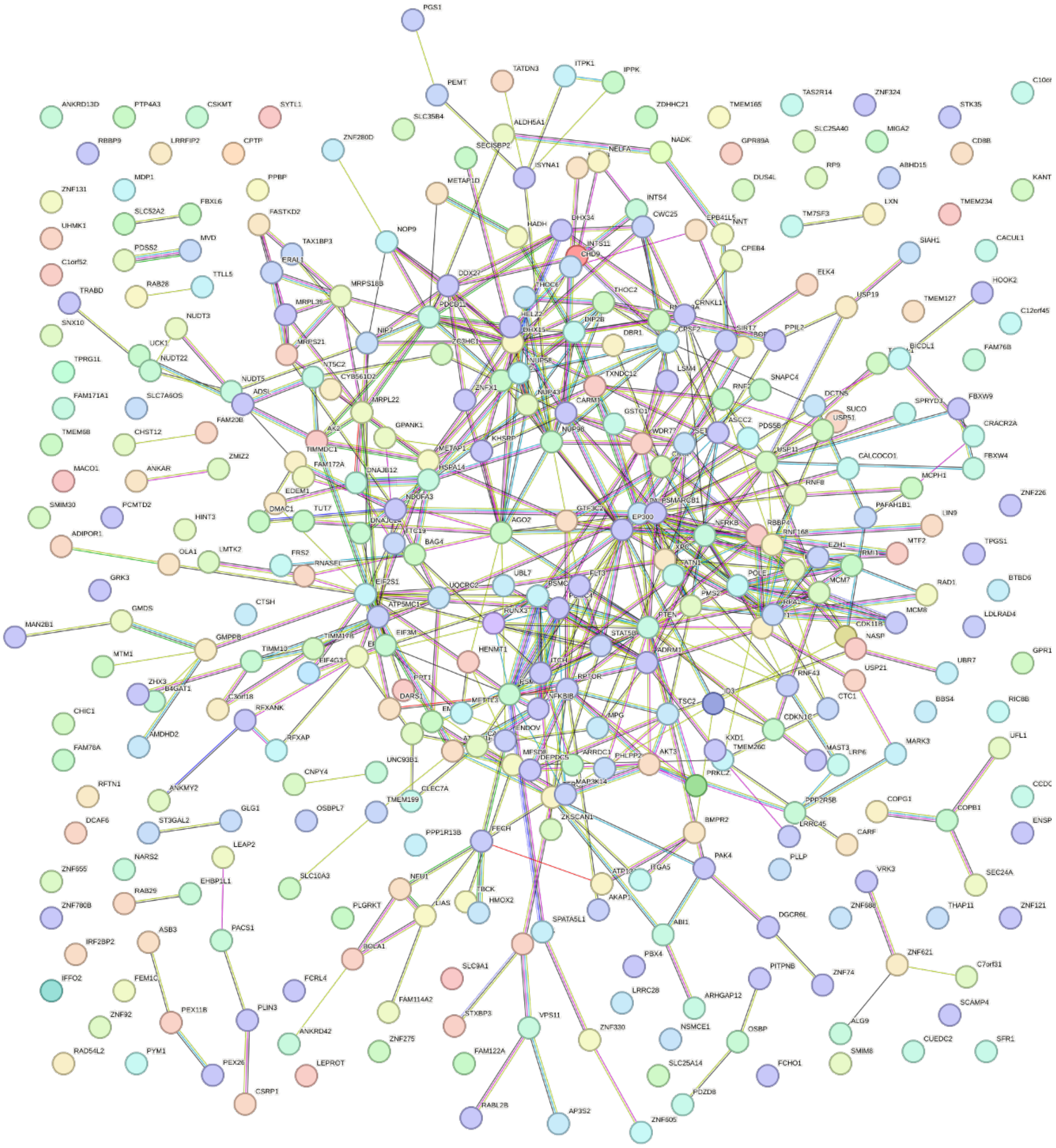

Figure S1. Protein-protein interaction (PPI) network of Differential Connectivity (DC) features. Network visualization of the 393 DC features identified in the T-cell activation dataset, generated using the STRING database. Nodes represent proteins, and colored edges represent known physical or functional interactions. The resulting network exhibits significantly more interactions than expected by chance (493 interactions observed vs. 392 expected; $p = 5.56 \times 10^{-7}$). This significant enrichment confirms that the features identified by MOSAIC are not randomly distributed but form a biologically coordinated regulatory network.

bioRxiv preprint doi: https://doi.org/10.64898/2026.02.10.705077; this version posted February 27, 2026. The copyright holder for this preprint (which was not certified by peer review) is the author/funder, who has granted bioRxiv a license to display the preprint in perpetuity. It is made available under aCC-BY 4.0 International license.

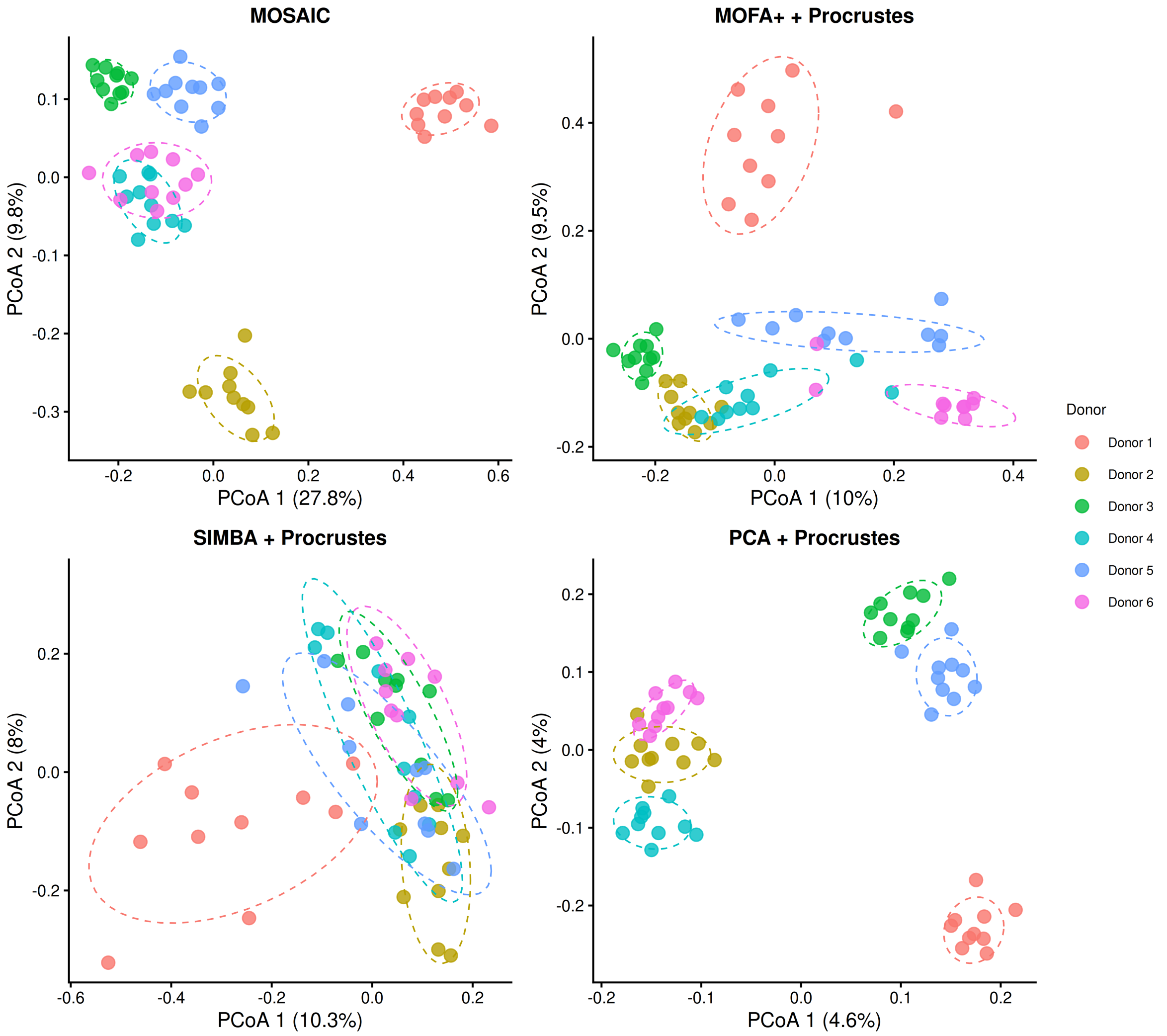

Figure S2. PCoA visualization of cross-sample embedding distances. Principal Coordinates Analysis (PCoA) of pairwise embedding distances at 200 cells per replicate for all four methods: MOSAIC, MOFA+ + Procrustes, SIMBA + Procrustes, and PCA + Procrustes. Points are colored by donor. Axis labels indicate the percentage of variance explained by each coordinate.
