# Supplementary material for: MOSAIC: A Spectral Framework for Integrative Phenotypic Characterization Using Population-Level Single-Cell Multi-Omics
Source: bioRxiv. 2026 Feb 12:2026.02.10.705077. Preprint. [Version 1] doi: 10.64898/2026.02.10.705077 (PMC12918988; doi:10.64898/2026.02.10.705077)
Supplement: Supplementary file 1 [file NIHPP2026.02.10.705077v1-supplement-1.pdf]

bioRxiv preprint doi: https://doi.org/10.64898/2026.02.10.705077; this version posted February 12, 2026. The copyright holder for this preprint (which was not certified by peer review) is the author/funder, who has granted bioRxiv a license to display the preprint in perpetuity. It is made available under aCC-BY 4.0 International license.

A Supplementary Methods

Biological Coherence Validation To validate that the MOSAIC embedding captures meaningful biological relationships, we utilized the multi-omic human prefrontal cortex (PFC) dataset [12]. We selected two representative samples (HCTXJ and 10280) and focused on three feature types: genes (RNA), chromatin peaks (ATAC), and transcription factors (TFs; derived from RNA expression). Features were pre-filtered to ensure data quality: genes were restricted to the top 6,000 highly variable features with total expression > 10 counts across all samples; TFs were filtered for total expression > 10 counts; and peaks were restricted to the top 1.5% most variable peaks. For each sample i, we used its specific feature embedding $\mathbf{E}_i \in \mathbb{R}^{F \times d}$. For every feature f, we defined its "Neighbors" as the set of features falling within a radius determined by the 10th percentile of all pairwise Euclidean distances in the embedding space. Conversely, we defined "Non-neighbors" as a random subset of 300 features located outside this radius. To quantify coherence, we computed the Pearson correlation between the original cell-level profiles (from $\mathbf{X}_i$) of feature f and its Neighbors versus Non-neighbors. These correlation distributions were aggregated across both samples to assess whether proximity in the MOSAIC embedding consistently reflects stronger functional co-variation in the original data.

Technical Robustness Validation To assess the stability of MOSAIC embeddings against sampling variation, we utilized the same two PFC samples described above. We performed a resampling experiment consisting of 100 iterations. In each iteration, the cells from each sample were randomly partitioned into 10 non-overlapping subsamples (pseudo-replicates), yielding a synthetic cohort of 20 subsamples $\{\mathbf{X}_{i,1}, \ldots, \mathbf{X}_{i,10}\}$ for both individuals $i \in \{1, 2\}$. We applied the complete MOSAIC framework to this cohort, generating 20 subsample-specific embeddings $\{\mathbf{E}_{i,k}\}$. We then computed pairwise dissimilarities between all subsamples using the squared Frobenius distance:

$$d(\mathbf{E}_a, \mathbf{E}_b) = \|\mathbf{E}_a - \mathbf{E}_b\|_F^2$$

This produced a 20×20 distance matrix for each iteration. To quantify robustness, we performed hierarchical clustering on this matrix and compared the resulting 2-cluster solution to the ground-truth donor labels. Performance was evaluated using the Adjusted Rand Index (ARI) for recovery of identity, and the Silhouette score for cluster cohesion. Additionally, we quantified the statistical distinctness of the donor-specific signatures using PERMANOVA (yielding R^2 and p-values) on the embedding distance matrices.

To quantify the recovery of biological identity, we performed K-means clustering ($k = 2$) on the subsample embeddings and compared the resulting partitions to the ground-truth donor labels using the Adjusted Rand Index (ARI). The ARI corrects the Rand Index (RI) for chance agreement and is defined as:

$$\text{ARI} = \frac{\sum_{ij} \binom{n_{ij}}{2} - [\sum_i \binom{a_i}{2} \sum_j \binom{b_j}{2}]/\binom{n}{2}}{\frac{1}{2}[\sum_i \binom{a_i}{2} + \sum_j \binom{b_j}{2}] - [\sum_i \binom{a_i}{2} \sum_j \binom{b_j}{2}]/\binom{n}{2}}$$

where n_{ij} is the number of subsamples common to cluster i and ground-truth class j, a_i and b_j are the sums of rows and columns of the contingency table, and n is the total number of subsamples. To evaluate the cohesiveness of the donor-specific signatures in the embedding space, we calculated the Silhouette Score. For a subsample i, the silhouette value is defined as:

$$s(i) = \frac{b(i) - a(i)}{\max\{a(i), b(i)\}}$$

where $a(i)$ is the average distance between i and all other subsamples in the same donor group, and $b(i)$ is the minimum average distance from i to subsamples in the other donor group. We reported the mean $s(i)$ across all subsamples. To establish a chance-level baseline for both metrics, we repeated the calculations using randomly shuffled subsample labels in each iteration.

Simulation of Multi-modal Differential Connectivity To rigorously evaluate MOSAIC against abundance-based methods, we developed a generative simulation framework based on Singular Value Decomposition (SVD). We simulated population-scale multi-omics data ($S = 20$ samples, evenly split into Conditions A and B) characterized by three distinct modalities (X, Y, Z) with feature spaces of size $p_x = 1{,}000$, $p_y = 800$, and $p_z = 600$ respectively (total $F = 2{,}400$ features). Each sample contained approximately 1,000 cells.

Generative Model For each sample i and modality m, the cell-by-feature matrix $\mathbf{X}_i^{(m)}$ was generated as $\mathbf{X}_i^{(m)} = \mathbf{U}_i \mathbf{\Sigma}^{(m)} (\mathbf{V}_i^{(m)})^\top$, where: (1) $\mathbf{U}_i \in \mathbb{R}^{n_i \times r}$ are sample-specific cell embeddings drawn from a

bioRxiv preprint doi: https://doi.org/10.64898/2026.02.10.705077; this version posted February 12, 2026. The copyright holder for this preprint (which was not certified by peer review) is the author/funder, who has granted bioRxiv a license to display the preprint in perpetuity. It is made available under aCC-BY 4.0 International license.

Gaussian mixture model ($k = 10$ clusters) to simulate cell-type structure in a latent rank $r = 10$; (2) $\mathbf{V}_i^{(m)} \in \mathbb{R}^{p_m \times r}$ are feature loadings, also drawn from a Gaussian mixture to simulate 10 feature modules shared by modalities; and (3) $\mathbf{\Sigma}^{(m)}$ is a diagonal matrix of singular values controlling signal strength.

Simulation Scenarios We selected 20% of features from each modality as DC features and generated three distinct signal types:(1) Pure Connectivity (Rewiring Only): For samples in Condition B, we permuted the rows of $\mathbf{V}_i^{(m)}$ corresponding to DC features. Crucially, permutations were constrained to swap features between distinct latent clusters, ensuring a maximal disruption of the local network topology rather than minor within-module variations. To remove abundance artifacts, we computationally rescaled the mean expression of these rewired features in Condition B to match the global mean of Condition A ($E[\mathbf{X}_B] = E[\mathbf{X}_A]$).(2) Confounded Signal (Rewiring + Mean Shift): We applied the same inter-cluster row permutation to $\mathbf{V}_i^{(m)}$ for Condition B but retained the resulting shifts in mean abundance, mimicking a scenario where rewiring induces expression changes.(3) Mean Shift Only (Differential Abundance): No connectivity changes were applied ($\mathbf{V}_A = \mathbf{V}_B$). Instead, the abundance of the selected features in Condition B was multiplied by a fold-change factor of 2.5.

Robustness and Benchmarking To test robustness, we introduced non-linear technical batch effects ($B = 4$ batches). For each feature j in batch $b \in \{1..4\}$, we applied a multiplicative scale $\alpha_j \sim \mathcal{N}(1.2, 0.1)$ and an additive shift $\beta_j \sim \mathcal{N}(10, 0.2)$ that was scaled linearly by batch index ($\beta_{j,b} = \beta_j \times b/4$), creating a complex, non-linear distortion. We further evaluated performance under varying signal-to-noise ratios by scaling the diagonal elements of $\mathbf{\Sigma}^{(m)}$. For the Strong signal scenario, singular values were linearly spaced from 10 to 1; for Medium and Weak scenarios, these values were scaled down to ranges of 5–0.5 and 0.1–0.01, respectively. Performance was quantified using the Area Under the Precision–Recall Curve (AUPRC).

T-Cell CITE-seq Data Analysis and Differential Connectivity Feature Identification For the real-data DC analysis, we utilized a public CITE-seq dataset (paired RNA + ADT) characterizing the immune response to vaccination in 8 donors (pre- and post-vaccination) [13]. We filtered for CD4+ Naive T-cells and applied MOSAIC to the $S = 16$ samples (8 donors × 2 timepoints). To ensure data quality and embedding stability, we pre-filtered the dataset to retain only genes and proteins with a total expression/abundance count > 3 across all samples. We then applied the MOSAIC framework to generate the feature × sample embedding tensor $\mathcal{E}$ and identified DC features as described above. To validate the stability of feature abundance (Figure 4C), we performed a pseudobulk analysis. Single-cell expression counts were aggregated by donor to compute the mean expression for each sample, and statistical significance between Day 0 and Day 7 was assessed using a T-test. To visualize the geometric shift in a feature's functional context (Figure 4D), we extracted the sample-specific embedding vectors for the target feature (e.g., STAT5B) from $\mathcal{E}$ and applied Multidimensional Scaling (MDS) to project these high-dimensional vectors into a 2D space.For the quantitative validation of rewiring (Figure 4B), we calculated the Jaccard Index of neighbor overlap. We first computed a consensus embedding for Day 0 (averaging $\mathbf{E}_i$ for all $i \in$ Day 0) and Day 7. For each feature f, we identified its set of top 20 nearest neighbors in the Day 0 ($\mathcal{N}_f^{(0)}$) and Day 7 ($\mathcal{N}_f^{(7)}$) spaces. The Jaccard Index was computed as $J = |\mathcal{N}_f^{(0)} \cap \mathcal{N}_f^{(7)}| / |\mathcal{N}_f^{(0)} \cup \mathcal{N}_f^{(7)}|$. Network plots (Figure 4E) were generated by visualizing these specific neighbor sets.

PFC Multi-omic Analysis and Subgroup Characterization We analyzed a public multi-omic cohort of human prefrontal cortex (PFC) tissue comprising 30 donors (18 HIV+, 12 controls). We specifically isolated L2/3 inhibitory neurons and, to ensure robust statistical modeling, excluded samples with fewer than 10 cells. Feature spaces were defined rigorously for both modalities: for snRNA-seq, we selected the top 10,000 highly variable genes with a total expression count > 3 across all samples. For snATAC-seq, we computed gene activity scores using the `Signac :: GeneActivity` function and retained the top 3,000 variable features with a total activity count > 5. For subgroup validation, we applied the MOSAIC pipeline to the full cohort of 30 donors, quantifying module activity using `Seurat::AddModuleScore`. For de novo discovery (Fig. 5D–H), we restricted the unsupervised clustering to the 18 HIV+ samples, identifying two distinct subgroups: "HIV-Group1" ($n = 10$) and "HIV-Group2" ($n = 8$). We characterized the molecular divergence between these groups by performing differential expression (DE) on RNA profiles and differential accessibility (DA) on ATAC gene scores using MAST. Functional interpretation was performed via Reactome pathway enrichment analysis on the set of genes statistically upregulated in HIV-Group1.

bioRxiv preprint doi: https://doi.org/10.64898/2026.02.10.705077; this version posted February 12, 2026. The copyright holder for this preprint (which was not certified by peer review) is the author/funder, who has granted bioRxiv a license to display the preprint in perpetuity. It is made available under aCC-BY 4.0 International license.

B Supplementary Figure

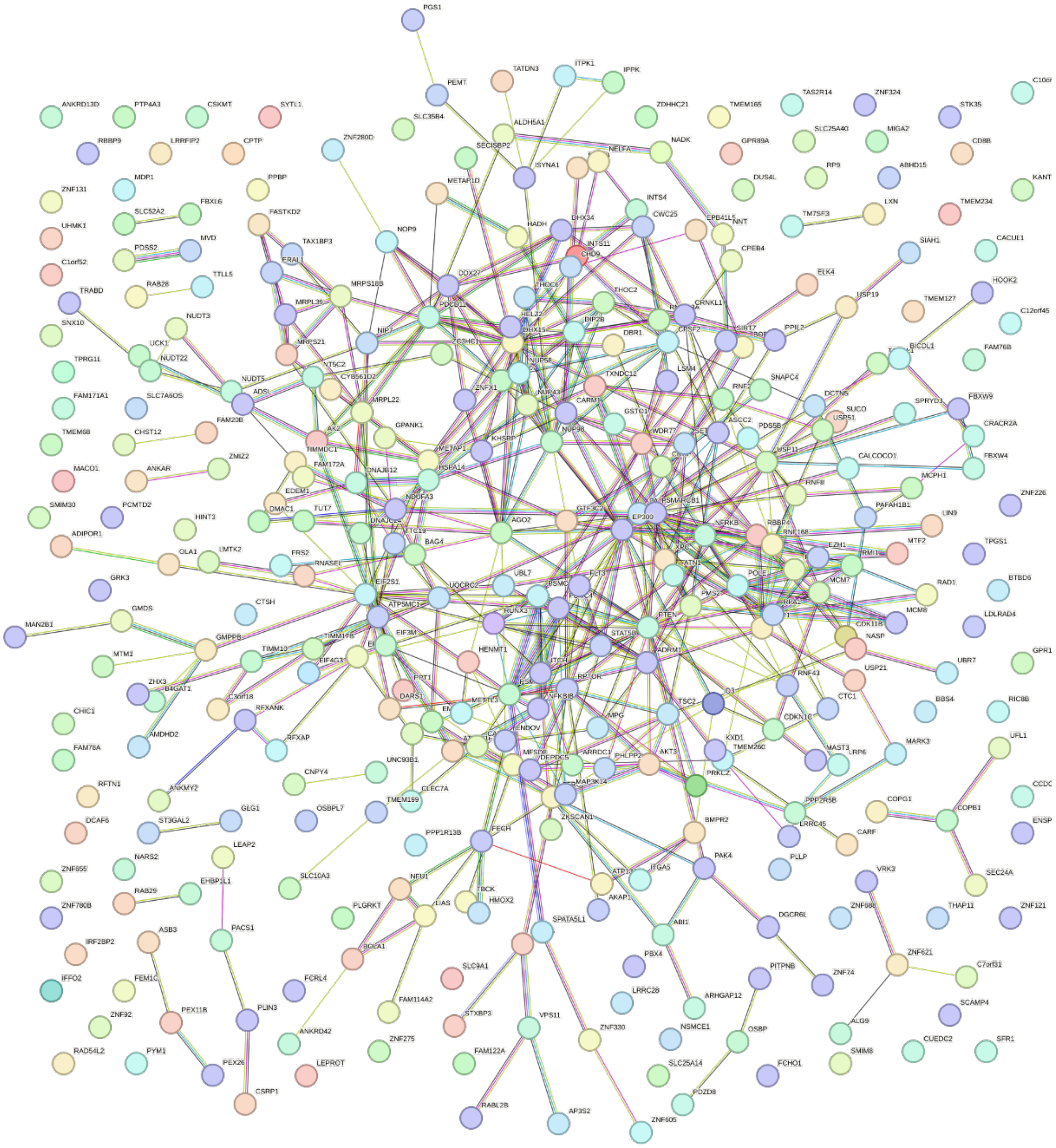

Figure S1. Protein-protein interaction (PPI) network of Differential Connectivity (DC) features. Network visualization of the 393 DC features identified in the T-cell activation dataset, generated using the STRING database. Nodes represent proteins, and colored edges represent known physical or functional interactions. The resulting network exhibits significantly more interactions than expected by chance (493 interactions observed vs. 392 expected; $p = 5.56 \times 10^{-7}$). This significant enrichment confirms that the features identified by MOSAIC are not randomly distributed but form a biologically coordinated regulatory network.
